# Protein Folding and Mechanisms of Proteostasis

**DOI:** 10.3390/ijms160817193

**Published:** 2015-07-28

**Authors:** José Fernando Díaz-Villanueva, Raúl Díaz-Molina, Victor García-González

**Affiliations:** Facultad de Medicina, Universidad Autónoma de Baja California, Mexicali, Baja California 21000, México; E-Mails: jdiaz92@uabc.edu.mx (J.F.D.-V.); rauldiaz@uabc.edu.mx (R.D.-M.)

**Keywords:** proteins, folding, proteostasis, misfolding

## Abstract

Highly sophisticated mechanisms that modulate protein structure and function, which involve synthesis and degradation, have evolved to maintain cellular homeostasis. Perturbations in these mechanisms can lead to protein dysfunction as well as deleterious cell processes. Therefore in recent years the etiology of a great number of diseases has been attributed to failures in mechanisms that modulate protein structure. Interconnections among metabolic and cell signaling pathways are critical for homeostasis to converge on mechanisms associated with protein folding as well as for the preservation of the native structure of proteins. For instance, imbalances in secretory protein synthesis pathways lead to a condition known as endoplasmic reticulum (ER) stress which elicits the adaptive unfolded protein response (UPR). Therefore, taking this into consideration, a key part of this paper is developed around the protein folding phenomenon, and cellular mechanisms which support this pivotal condition. We provide an overview of chaperone protein function, UPR via, spatial compartmentalization of protein folding, proteasome role, autophagy, as well as the intertwining between these processes. Several diseases are known to have a molecular etiology in the malfunction of mechanisms responsible for protein folding and in the shielding of native structure, phenomena which ultimately lead to misfolded protein accumulation. This review centers on our current knowledge about pathways that modulate protein folding, and cell responses involved in protein homeostasis.

## 1. Introduction

One of the keystones that has been considered to drive the evolution of organisms, relies on the capacity to detect, respond, and adapt to various stressors in the environment through cellular defensive mechanisms that protect the entire organism and maintain its capacity to grow and reproduce [1]. For instance, establishment of preformed enzyme complexes for cell function has been proposed as a mechanism used by cells to rapidly respond to homeostasis unbalances [2,3], whereas pathways that regulate the conservation of protein folding have a key role [4].

Function in proteins largely depends on the acquisition of specific three-dimensional structures by folding at physiological time scales. Cells have developed highly controlled mechanisms to maintain native protein folding, which include detail three-dimensional structure patterns and specific disordered domains. Notwithstanding, although the process is extremely efficient, there is always the possibility that this accurate mechanism fails, and, in consequence, finding a protein folded into a non-native state becomes a reality [5]. In this case, the structural stability of proteins depends largely on hydrophobic residues being oriented towards the protein core. This has allowed evolution to develop a conserved warning system, in which the exposure of protein hydrophobic regions is recognized as a molecular pattern associated with the presence of cytotoxicity [6]. For instance, oligomerization preceding amyloid fibril formation has been associated with cytotoxic effects, which may arise from their misfolded conformation in which hydrophobic side chains are exposed to the cytosol [7,8]. Cytotoxic effects could be triggered by interactions among hydrophobic regions of proteins exposed to aqueous microenvironment with cellular biomolecules, such as other proteins, nucleic acids, or lipid membranes [9,10].

Cells modulate protein folding and protein degradation through extensive signaling networks to avoid misfolded protein accumulation [11]. Likewise, cells must not only promote accurate protein folding but also prevent the accumulation of misfolded species that may arise from translation errors, and synthesis of aberrants mRNAs [12]. Proteomes have been described as multifaceted and constantly evolving entities [13]. Then, the understanding of the quality control checkpoints, which are performed during normal cell physiology, and those that are activated during protein stress, including the stress responses, function of catabolic machineries, and systems of communication among molecules and organelles, are subject to an extensive research.

Most of the signaling proteins used by eukaryotic cells to communicate with their environment are assembled in endoplasmic reticulum (ER). Transmission and management of information through proteins is crucial for the homeostasis of the organisms, regulating mechanisms such as cell cycle, apoptosis, and cell growth [14]. Loss of protein structure can arise from alterations in diverse stages during protein synthesis, the degradation process, or changes in concentration of metabolites in the cell environment. Given the central role of protein folding in biology, it is interesting to think that a misfolding can lead to dysfunction, modifying the cellular mechanism in which the protein is involved. In fact, many chronic-degenerative diseases are associated with an aberrant protein folding, modifying the condition of protein homeostasis (proteostasis) to cause situations such as ER stress [15,16,17].

**Figure 1:**
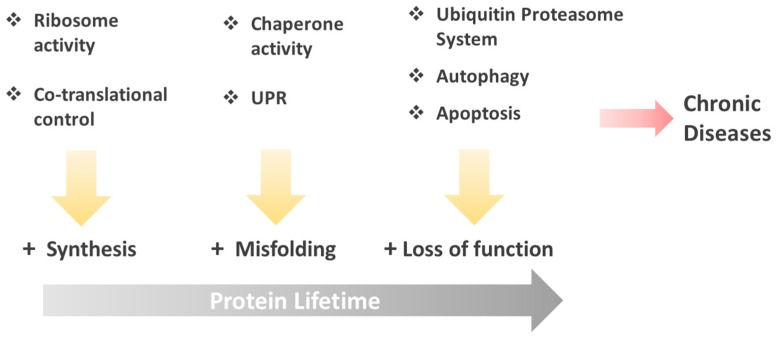
Molecular and cellular mechanisms to maintain native protein structure. The vertical arrows indicate the mechanisms which cells employ to counteract alterations in native protein structure, during their lifetime (horizontal arrow). When the cellular machinery designed to control widespread protein misfolding or aggregation fails, apoptosis ensues. When apoptosis cannot restrict the systemic spread of protein misfolding, chronic diseases originate (red arrow).

Several points of interconnection within pathways responsible for maintaining the cell functions converge on mechanisms of protein folding, and therefore, on the conservation of native three dimensional structure of proteins. In this paper, the main focus will rely on the analyses of mechanisms related with the chaperone protein function and unfolded protein response (UPR) pathways, in addition to strategies developed by cells such as the spatial compartmentalization of protein folding, protein degradation by proteasomes, and autophagy; all of these mechanisms connected with the objective to conserve the proteostasis (Figure 1). Misfolding condition seen through various topics is a fundamental field in the understanding of protein nature, and cells devote many resources for its regulation. Therefore, the understanding of these molecular mechanisms is a cornerstone for optimum pharmacological treatments of diseases associated with protein misfolding.

## 2. Protein Structure

### 2.1. Protein Folding

Cell functions need to be regulated with high levels of efficiency, a condition under strict control is the acquisition and maintenance of the native three-dimensional structure of proteins. A protein acquires its functional structure through a folding phenomenon, in which its amino acid sequence acquires the minimum energy conformation [18]. For folding into a native state, unfolded polypeptide chains require the intervention of weak interactions. Driven by hydrophobic interactions, a polypeptide chain begins to fold when it is present in an aqueous environment after synthesis, and rapidly becomes a molten globule followed by an important release of latent heat. Stabilization of the molten globule is achieved mainly through the distribution of hydrophobic residues away from the bulk water. On the other hand, because the polar residues contained in a protein develop hydrogen bonds with the water network as well as with each other, α-helices and β-sheets can be formed when bonds switch between molecules. It has been calculated that such bonds might be in the order of 10^−12^ s, very similar to those we find in water itself [19].

The random equilibrium can be shifted toward one of these conformations by means of two stages: a fast stage, during which the unfolded polypeptide becomes a molten globule; and a slow stage, in which the molten globule slowly transforms into a fully folded native state [19]. Considering that the native state is located at the minimum of the folding funnel, it indicates that this region is the most thermodynamically stable configuration of the polypeptide chain [20].

Chains of amino acids must acquire their three-dimensional structures in very short time scales, which is a requirement to operate within a highly concentrated cellular environment. Although varied and intricate structures of globular proteins are encoded by their amino acid sequences, and these molecules have an intrinsic ability to fold spontaneously [21], under normal physiological conditions, some proteins do not fold into globular structures [22,23], and a negative effect on cell physiology might occur.

### 2.2. Allosteric Approach to Proteins

Proteins have been visualized as dynamical objects that interconvert between a diversity of structures with varying energies, rather than static sculptures [24]. In this sense, these biomolecules can be physicochemically described not as single structures but, rather as conformational ensembles with dynamic distributions of states, which can change under different micro-environmental conditions [25]. This feature represents a possible association with a new base to understand the allostery, a phenomenon related in the first case to describe the dynamic modularity of proteins, and subsequently in the possible understanding of cellular dynamics.

While the function of several proteins has been characterized from an *in vitro* approach, information regarding the influence of the highly crowded cellular environment, or the effect of metabolites and molecules on protein function is not complete. Membrane systems, multi-enzyme complexes, macromolecular structures, and metabolite concentrations should have a direct influence on protein function and, ultimately in the regulation of cell physiology [26]. For example, perturbations in the native three-dimensional structure of proteins does not produce isolated effects on the single protein but, rather could affect the assembly of macromolecular structures, modifying the surrounding microenvironment [27].

Traditionally, the allosteric phenomenon has been associated with conformational and functional transitions on individual proteins; for instance, regulating many features of enzyme performance. However, some authors have extended this concept to include the impact of protein conformational perturbations on cellular function [25,28]. It has been proposed that multi-scale organization across different levels provides a feedback regulation on specific proteins, and collectively on cell signaling pathways. Through this perspective, proteins perform their functions by highly interconnected cellular pathway linkages. Therefore, changes in their conformation by allosteric effects, could propagate across networks of multi-complex assemblies [25,29]. Then, allostery is proposed as a fundamental regulatory device of the cell, used to modulate its activity in response to external and internal stimuli.

Depending on the stimuli that cells receive, multi-scale spatial cellular organization could shape signaling, and coordinate cellular behavior, through protein clusters [30]. The coordination of the activities and responses of the cell to its environment could emerge from pre-organized assemblies across the cell with different length scales. Together, these conditions provide a framework of a spatial organization of signaling cascades, where signaling could proceed through intermolecular interactions among and within clusters of proteins [2]. Spatial structure in cells offers a glimpse into the high organization in all levels, from small molecular complexes and assemblies, to local nanoclusters, and micrometer scales between cells [31]. Likewise, spatial structure of cell signaling systems could be described in function of dynamic allosteric interactions within and among distinct spatially organized transient clusters of biomolecules [2].

The existence of intrinsic dynamism as well as static properties of biological systems has been well established, and although instance the inherent fluctuations within the proteins have been explored in a specific view, and linked with processes ranging from the acquisition of the three-dimensional structures to the mechanism of enzymatic action [32,33]. In this focus, protein structure can be understood as a factor that exerts influence in its surroundings and ultimately in the spatial organization of the cell. Additional to their functional native structures, proteins or specific regions in them, acquire other conformations including disordered and partially ordered conformations [34].

### 2.3. Partially Folded States in Proteins

While the partially folded states in proteins are difficult to conceptualize and their experimental study is challenging, a wide variety of roles for protein structure disorder has been proposed [35,36,37]. Disordered proteins are visualized as dynamic assemblies, wherein the atom positions and Ramachandran angles axis vary significantly over time. Disordered proteins differ from their counterparts structured in their dynamics. Intrinsically disordered proteins (IDP) represent a challenge to the previously described structure–function concept, in this case the dominant feature is lack of persistent secondary and tertiary structure on these proteins [38,39,40].

The large degree of conformational sampling for IDP gives them significant conformational entropy, which can be restricted by intra- and inter-molecular interactions. Moreover, it has been suggested that loss of conformational entropy upon ligand binding originates a weaker binding for IDP, undergoing disorder-to-order transitions in secondary structure upon ligand interactions [41]. Structural disorder may span from short segments in specific domains to entire proteins [42], indeed there is experimental evidence of random conformations in 1539 domains of 694 proteins deposited in a specialized database (DisProt) [43]. The preponderance of disorder in proteins involved in signaling networks within higher eukaryotes in comparison with constitutive metabolic proteins or in bacteria [44], suggests a specific function for IDP, which might be linked to molecular recognition [45,46].

The importance of disorder in proteins is self-evident, as a large portion of molecular interactions depend on the complementary interaction between structurally organized proteins and IDPs. This structural condition could confer diverse advantages, such as rapid and specific binding, thus the ability to carry out some other functions [47]. Interactions among the motifs are usually weak, transient, and cellular-milieu dependent [48,49]. For instance, the disordered state is present in proteins associated with transcription, signaling, phosphorylation, RNA processing, ubiquitination, ion transport, cytoskeletal organization, cell cycle control, and other highly regulated biological mechanisms [50,51]. From an evolutionary point of view, it appears that intrinsic disorder in proteins might have been the driving force behind many of the adaptability processes found in proteins [5,52].

The relevance of the absence of rigid structures in proteins in the establishment of communication between protein networks is evident in the cases of the so-called, guardian of the genome, protein p53 and the proto-oncogene, c-myc, which have long disordered regions [53,54]. The use of IDPs or unstructured domains in proteins can also prove be deleterious as many of the proteins that are involved in the most common misfolding diseases are intrinsically disordered.

Likewise, specific domains or complete proteins lacking defined tertiary structures are known to have the fingerprint to undergo disorder-to-order transitions upon binding to specific or multiple partners [55,56]. This ability allows the concept of protein disorder to be proposed as an important feature in the capability of proteins to present regions with switch properties [57,58,59], condition that could modulate the function, and respond to specific changes in the surrounding microenvironment. The basic properties of a switch mechanism must be based on the equilibrium between high specificity and weak affinity, accompanied by a large conformational entropy decrease [60].

Disorder-to-order transitions in proteins playing normal switching roles in the cell might become distorted and therefore abolish or transform the normal protein-protein language into an aberrant one. This is the case for α-synuclein, an important protein found in Lewy bodies in the brain of patients affected with Parkinson disease [61]. In the situation of prion diseases, the PrP protein was isolated from amyloid plaques, in which a clear conformational change in secondary structure from α-helix into β-sheet following a templating mechanism, was recognized as the process that could cause the misfolding phenomenon [62].

### 2.4. Amyloid Focus

Cells have developed effective strategies to maintain native protein folding, either through an ordered three-dimensional structure or the use of disordered domains [37]. However, the amyloid fibrils which represent self-associated species of peptides and proteins [63], considering their remarkable structures and properties, are of particular interest. Misfolding of normally soluble peptides and proteins has been associated with about 50 disorders with a multitude of different symptoms, in which mechanisms of non-native interactions could form aggregates, including the archetypal amyloid-like fibril [64,65].

Amyloid state has been proposed as a generic condition, being accessible to different polypeptide chains, and, unlike the native state, its essential three-dimensional architecture is not encoded by the amino acid sequence [66]. Determined through X-ray fiber diffraction studies, amyloid fibrils show a common cross-β pattern that is indicative of a structure mainly β-strand, being oriented perpendicularly to the fibril axis [63,67,68]. The cross-β architecture provides great stability to the fibrils because it allows the formation of a continuous arrangement of hydrogen bonds [69], complementary steric interactions. and the formation of a repetitive structural pattern.

Amyloid structures are relevant not only in the context of disease, but also because their occurrence challenges in many ways our current understanding of the nature, structure, and evolution of the functional state of proteins [70,71,72,73]. From a wide range of experiments on peptides and proteins, we now know that the formation of amyloid structures is not a rare phenomenon associated with a small number of diseases, but rather that it reflects a well-defined structural folding of proteins [66,70,74]. Functional amyloids have been described in organisms such as bacteria [75,76], fungi [77,78,79], insects [80,81], and mammals [82,83]. Specific cellular function related with the formation of amyloid-like fibril structures has been described, suggesting that amyloid deposition can be a common property of the polypeptide chains. The difference between functionality and apparition of toxicity may depend on the regulatory mechanisms that cells have evolved to modulate their formation.

The transition of a protein from its functional native conformation to amyloid fibrils is a complex phenomenon that results from the interplay between various elementary processes and a rearrangement in molecular interactions on precursor proteins. As aggregation produces arrangements of different oligomers, various types of toxic species can be expected considering a mixture of molecular populations, indeed almost any misfolded specie is likely to have the potential to generate at least some level of toxicity [84].

**Figure 2:**
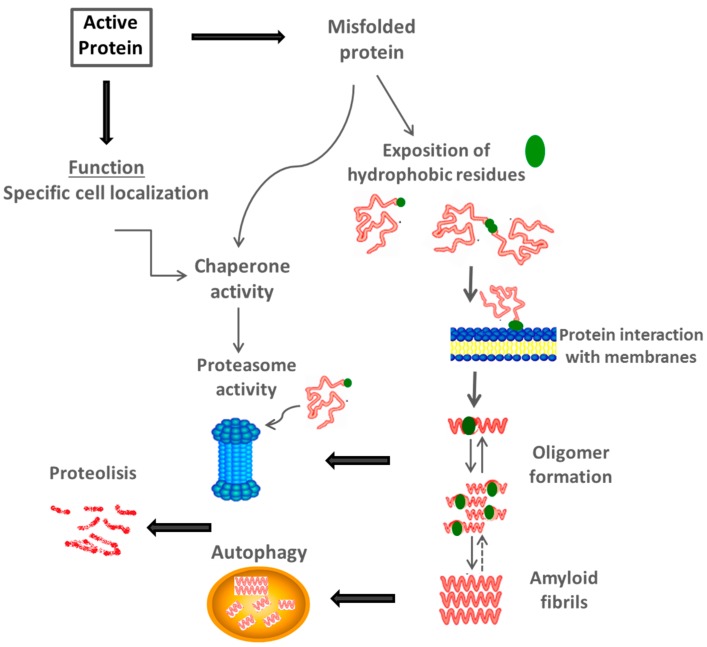
Cell mechanisms that control protein structure. Alterations in protein structure during folding can result in anomalous interactions with inner membranes through the exposition of hydrophobic surfaces. Cellular mechanisms, such as proteasome activity and autophagy, could reduce toxic effects of these molecules, and ultimately prevent cell damage. Likewise, these processes occur during physiological protein turnover (Adapted from [6]).

A panorama has emerged over the past 15 years that mature amyloid fibrils are not primary toxic compounds, but molecular species which are precursors to their formation, such as oligomers. Possibly in the primary mechanism of fibril formation, the exposure of hydrophobic residues is critical for the nucleation process, a condition that would favor the formation of intermolecular contacts, offering thermodynamic stability for the amyloid structure (Figure 2) [85]. Although many of the protein characteristics that preclude amyloid formation are encoded by their amino acid sequences, the elucidation of this code has enabled the identification of factors that determine the aggregation propensity of proteins [86,87].

Under the exposition of hydrophobic patches to the aqueous medium, proteins might find a stable conformation to decrease major toxic effects through the formation of amyloid structures. Misfolded proteins tend to expose hydrophobic regions, which are normally hidden from the aqueous environment, either by folding characteristics, insertion into membranes or by the support of accessory molecules. This exposure may cause cytotoxic effects, especially associated to anomalous interaction with lipid membranes, in turn causing serious damage to the bilayer. Activation of the innate immune response leading to inflammation has been described as one of the first mechanisms of toxicity associated with prions, amyloid β (Aβ) peptide, amylin, and other proteins [88].

Likewise, cell damage and death are likely to become widespread only when misfolded and aggregation-prone species, reach levels that can overwhelm the defensive housekeeping systems [37,38,89,90]. This breakdown of proteostasis [89,91] can lead to a situation that is known as protein metastasis, in which initial aggregation events trigger a cascade of pathological processes that could mark the progression of disease [92].

## 3. Mechanisms to Conserve Protein Structure

During their functional lifespan, proteins can undergo deviations from their native three-dimensional structure. In order to maintain the native protein folding, cells have developed various strategies to accomplish this task, through sophisticated chaperone and quality control networks that can resolve damage at the level of protein, organelle, or cell (Figure 1). For instance, on the smallest scale, the integrity of individual proteins is monitored during their synthesis in ribosomes, and coupled with co-translational chaperone function. On a larger scale, cells use compartmentalized defenses and networks of communication capable of signaling between cells, and so respond to changes in the proteome homeostasis [13]. Together, these layered defenses help protect cells from alterations in protein folding and degradation, avoiding the appearance of misfolded proteins and deleterious events [40].

The organization and evolutionary dynamics of the proteome provide a line of defense in protein quality control, wherein sequences have evolved to avoid aggregation, most proteins tend to be short and fold efficiently. This allows the energy-dependent chaperone systems to preferentially protect long, aggregation-prone, but functionally important proteins. This condition justifies the need for energetically costly quality control mechanisms to secure protein folding [93,94].

### 3.1. Chaperone Protein Function

Molecular chaperones are central elements of these quality control systems, as they facilitate protein biogenesis by assisting polypeptide folding, translocation, and assembly of newly made proteins in the crowded cellular environment [95,96]. While denatured proteins can refold *in vitro* without auxiliary factors, in the crowded molecular cell environment highly specific folding machinery is required, considering that several billion of protein molecules could exist at concentrations from 50 to 300 mg/mL [97,98].

The recognition of partially unfolded proteins through exposed hydrophobic patches [99,100] allows the binding of molecular chaperones to a large variety of different protein folding intermediates, this condition prevents non-specific protein interactions (Figure 2). Likewise, this chaperone function is associated with proteins during conditions such as the denaturation processes, oligomeric assemblies, proteins that have been translocated to a cell compartment, and during the assistance of proteolytic degradation [101]. This offers protection of biological molecules against stress-induced unfolding, along with support of the proteasome quality-control machinery which recognizes unfolded molecules and subsequently degrades them [102,103,104]. Thus, molecular chaperones inhibit the formation of protein aggregates maintaining proteins in a native conformation [105,106].

Molecular chaperones, also known as stress proteins or heat shock proteins (Hsp) are classified into families on the basis of molecular weight monomers (Hsp40, Hsp60, Hsp70, Hsp90, Hsp110 and small Hsp), although most of them exist as oligomers. Based on the sort of interaction with client proteins, chaperones are also classified in holdases, foldases, and disaggregases. Holdases are ATP-independent proteins, which can recognize and stabilize partially folded proteins, preventing their aggregation and presenting client proteins to foldases. The ATP-dependent foldases are directly involved in protein folding. Disaggregases are also ATP-dependent, which disaggregate the client protein aggregates and transfer the partially folded proteins to a holdase and/or foldase [107,108].

Chaperones Hsp70, Hsp90 and Hsp60 recognize hydrophobic side chains exposed to water environment in unfolded proteins. This activity is achieved with the support of ATP-independent chaperones, and the small Hsp (Hsp10 and Hsp40), which function as holdases, and are denominated co-chaperones. Binding of chaperones to these hydrophobic regions temporarily blocks protein aggregation, while ATP hydrolysis is important to allow folding of client unfolded proteins [101,109]. Although Hsp70 and Hsp60 share this mechanism to perform their function, they show a significant difference because Hsp70 releases the client protein for its folding in solution, while Hsp60 form a cylindrical multimeric complex within which the folding of client protein occurs. The Hsp10 functions as a lid of the Hsp60 complex to close its cavity, whereas the hydrolysis of ATP induces conformational changes in the inner surface of the cylindrical complex, allowing the folding of client protein [110]. Hsp60 with the support of its co-chaperone Hsp10 is responsible for folding and the refolding ATP-dependent, mechanism that allow up to 30% of folding in cells [111].

Chaperones of the Hsp70 family are regulated by co-chaperone Hsp40, preventing the aggregation of unfolded proteins. This process is involved in folding of proteins that have been translocated to a cellular compartment, in turn regulate the heat shock response, and disassemble multimeric protein complexes [111]. Hsp70 and Hsp90 are present in abundance within the ER.

Immunoglobulin binding protein (BiP) also known as GRP78 is the major Hsp70. Specifically, BiP binds short stretches of hydrophobic residues exposed to the aqueous environment in unfolded proteins, its activity reduces the effective concentration of aggregation-prone sequences. BiP allows progression of folding through release and rebinding cycles in unfolded protein substrates, consuming ATP during the process. With each release cycle, the client protein has the opportunity to fold. In this sense, folding competes with chaperone binding, and both processes offer the thermodynamic stability to proteins to acquire native structure [15,112].

Heat shock response (HSR) is coupled with the disposition of denatured proteins in the cytosol [113,114]. The transcription factor heat shock factor 1 (HSF1) is a key molecule in the coordination of HSR. This transcription factor is activated during cellular stress induced by the presence of unfolded proteins, and leads to the transcription of chaperones and other genes that modulate the folding. Under normal conditions, HSF1 in a monomeric inactive form is bound to the cytosolic Hsp70 and Hsp90. However, when the load of unfolded proteins increases, these chaperones will dissociate from HSF1 and be recruited to unfolded protein localization. Then, HSF1 trimerizes and translocates to the nucleus, where it is post-translationally modified by phosphorylation, and activates the transcription of HSP genes by binding to DNA containing heat shock elements [115].

Although, most knowledge about chaperone function comes from studies of chaperones with well-defined three-dimensional structures such as Hsp70 and Hsp90 [116], information regarding client-protein interactions which depend on large ATP-driven conformational rearrangements and interactions with co-chaperones is lacking [117,118]. Regions lacking defined three-dimensional structures in chaperones have shown to be critical for the function of certain chaperones, because they could modulate direct interactions with target proteins [116,119]. The lack of an ordered structure in regions of chaperones has also been suggested to be important for their ability to bind multiple aggregation-sensitive client proteins [35]. Disordered chaperones could represent a unique structure-function relationship, because their structural adaptability enables them to interact with structurally diverse targets through direct molecular contacts [116]. Disordered regions of proteins could assume diverse conformations upon binding to different partner proteins [35], and their chaperone functions could be extended.

### 3.2. Endoplasmic Reticulum Stress

Protein folding assistance through chaperones is complemented by the role of certain organelles. Considering that about one-third of the human proteome is synthesized in ER and transits to membrane compartments, the ER is an organelle that plays key roles in cell homeostasis, such as protein folding in the protein secretory pathway, lipid biosynthesis, and calcium storage. Lumen of ER is the major site for protein folding in the cell as it contains a variety of molecular chaperones and protein-folding enzymes [120]. Therefore, only properly folded proteins are packaged into ER vesicles for further transport to destination sites [121]. Proteins enter to ER as nascent, unfolded polypeptides at rates that can vary widely within the cell depending on the requirements of metabolic conditions [122]. When this machinery is overwhelmed by the increased demand of protein folding capacity, cells suffer a condition known as ER stress, a consequence of accumulation of unfolded or misfolded proteins in the lumen [15].

Through a network of intracellular signaling pathways that maintain the folding capacity [122], UPR is used to re-establish the homeostasis of protein folding function in ER [15]. UPR regulates translation and gene transcription to reduce the protein-folding load, in turn, increasing the folding capacity to contend with stress conditions. Distinct downstream signaling pathways are modulated for UPR signal transducers, three branches operate in parallel, whereas each branch is defined by a specific transmembrane ER-resident signaling component: IRE1 (inositol requiring enzyme 1), PERK (PKR-like ER kinase), and ATF6 (activating transcription factor 6). Synthesis of ER resident chaperones and folding catalysts, is induced to increase the folding capacity; and global mRNA translation is attenuated to decrease the folding load [123]. Likewise, through a process called ER-associated degradation (ERAD) misfolded proteins could be retained in the ER and retrotranslocated into the cytosol for proteasomal degradation [124]. UPR operates within the context of a translocation machinery that is compartmentalized between cytoplasm and ER [125].

ATF6 is a transcription factor that is initially synthesized as an ER-resident transmembrane protein bearing a large ER-luminal domain. Upon accumulation of unfolded proteins, ATF6 is packaged into transport vesicles that pinch off the ER and deliver it to the Golgi apparatus [126]. ATF6 is processed by two proteases, S1P and S2P (site-1 and site-2 protease), that sequentially remove the luminal domain and the transmembrane anchor, respectively [127,128]. The liberated N-terminal cytosolic fragment, ATF6 (N), then moves into the nucleus to activate UPR target genes involved in protein folding, such as BiP, the protein disulfide isomerase, and GRP94 (glucose-regulated protein 94), a chaperone of the Hsp90 family [14].

The second branch of the UPR is coordinated by PERK. When it is activated upon sensing ER stress, PERK oligomerizes and phosphorylates itself and the ubiquitous translation initiation factor eIF2α, then indirectly inactivates eIF2 and inhibits the global translation of mRNA, conditions that ameliorate the ER stress [14]. Concomitantly, translation of the transcription factor ATF4 is induced, which promotes transcription of two important target genes, CHOP (transcription factor C/EBP homologous protein) and GADD34 (growth arrest and DNA damage-inducible 34). CHOP is a transcription factor that controls genes encoding components involved in apoptosis. GADD34 encodes a PERK-inducible regulatory subunit of the protein phosphatase PP1C that counteracts the action of PERK by dephosphorylating eIF2α [14,129,130]. Then, this pathway has an intrinsic feedback regulation.

**Figure 3:**
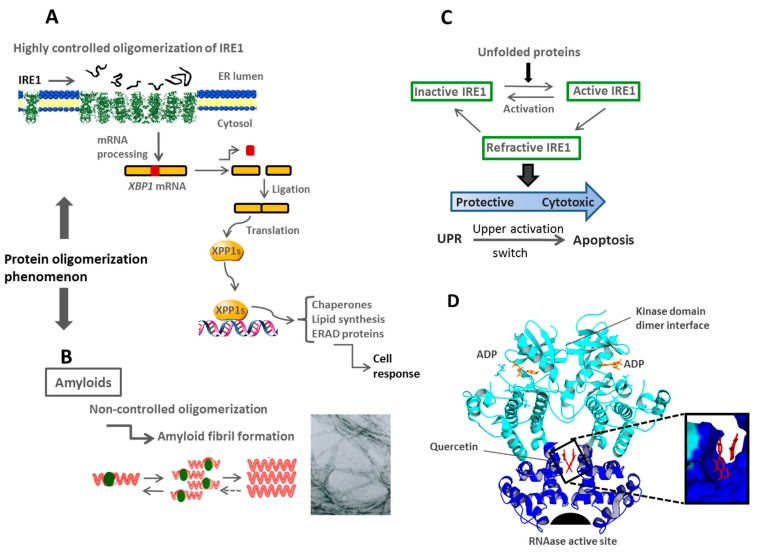
Activation of the inositol requiring enzyme 1 (IRE1) branch of the unfolded protein response (UPR) pathway is tightly controlled. (**A**) Schematic representation of IRE1 oligomerization and cellular response induced by unfolded proteins. The structure employed was obtained from the protein data bank (PDB) access code: 3fbv; (**B**) Uncontrolled protein aggregation in the formation of amyloid fibrils; (**C**) Effect of the overactivation of IRE1 branch on cellular homeostasis. Adapted from reference [141]; (**D**) Structural representation of IRE1 with a focus on the surface of interaction between monomers. *Inset:* sterol binding site, with two bound molecules of quercetin. Structure was obtained from PDB access code: 3LJ0.

The best-studied branch of UPR is IRE1, which transmits UPR signaling through a bifunctional transmembrane kinase/endoribonuclease that splices mRNA through a non-conventional mechanism. Binding of unfolded proteins triggers conformational changes following lateral oligomerization on the ER membrane, which in turn activates the IRE1 ribonuclease activity. Furthermore, IRE1 processes the mRNA of transcription factor XBP1 (X-box binding protein 1) [14] (Figure 3). This active, processed form of the transcription factor XBP1s controls expression of genes with X-box elements in their promoters, genes encoding ER chaperones and folding catalysts [131,132,133,134]. Additionally, the IRE1/XBP1 pathway is essential to activate genes which carry out ERAD functions [135], promoting the development of an elaborate ER response [136]. Active conformation of the kinase domain of IRE1 has been revealed through its crystal structure, to be an oligomeric assembly [122]. Indeed ribonuclease activity of IRE1 has been described to be proportional to the extent of IRE1 oligomerization [137].

UPR represents a focal point where different sources of stress converge, and stress signaling is coordinated within tissue hierarchies and further integrated [14]. Several activated transcription factors generated by UPR enter the nucleus and activate the production of their target genes, this mechanism establishes a feedback loop that relieves the ER stress by supplying more ER protein-folding capacity according to cell requirements [138]. In addition to linear information flow, the three branches of UPR transmit information to each other through a phenomenon known as cross-talk to fully integrate the signaling networks [139]. Nodes of interaction and communication between proteins required for cellular function should be highly regulated.

More experimental evidence is needed to fully understand specific thresholds necessary for the activation of stress signaling pathways, and in turn when homeostasis is reached again, the mechanisms that allow these responses to be turned off. For instance, IRE1 and PERK signaling duration is critical to determine the fate of cells during prolonged stress [140]. Therefore, when homeostasis cannot be re-established, UPR switches from a protective mechanism to a cytotoxic response [141], indeed UPR can function as an apoptotic executor which decreases cell viability (Figure 3).

Taking into account that ER must manage folding and modification of proteins in concentrations surpassing 100 mg/mL [142], recognition of unfolded proteins must be a highly precise mechanism to initiate the correct cellular response. Additionally, other mechanisms constitute a stress adaptation pathway that could reestablish homeostasis of proteins.

### 3.3. Spatial Compartmentalization of Protein Folding

Connections between loss of proteostasis, protein aggregation phenomenon, and conditions ranging from ageing to neurodegeneration, underscore the importance of the knowledge of the mechanisms that cells employ to manage protein misfolding [142]. For instance, it is estimated that up to 15% of nascent chains in human cells are co-translationally tagged for degradation, which emphasizes the importance of co-translational degradation in protein quality control at the ribosome [143,144]. Cells must not only promote accurate folding but also must prevent the accumulation of misfolded species that may arise from inefficient folding, errors in translation, and aberrant mRNAs [145].

An important condition to maintain the functionality of cells is associated with localization of misfolded or aggregated proteins into specialized compartments that are distinct from the organelles. Cytotoxicity is avoided through the confinement of misfolded proteins, aggregates, or amyloid like structures within appropriate and specific subcellular compartments, thus avoiding the subsequently nucleation of protein aggregates. In this sense, cells have developed mechanisms to solubilize and fold these proteins, when possible, leaving degradation in defined quality control compartments as a last-resort mechanism [146].

When quality control machineries fail, such as those previously mentioned, protein-controlled sequestration into specific compartments represents an alternative cellular defense against proteotoxic stress [147,148,149]. Upon proteasome impairment, misfolded proteins are distributed into spatially and functionally distinct compartments. Evidence has revealed a conserved sequestration of ubiquitinated proteins into membrane-enclosed juxtanuclear compartments, such as JUNQ (juxtanuclear quality control compartment). JUNQ is a cellular quality control space wherein soluble misfolded proteins accumulate for refolding or proteasomal degradation [150].

Under proteotoxic conditions in a cell, several chaperones and proteasome complexes can be located surrounding the JUNQ compartment, suggesting that JUNQ allows the concentration of misfolded proteins with chaperones, therefore increasing the probability of refolding, as opposed to simple uncontrolled degradation [151]. Substrates targeted to JUNQ are primarily soluble proteins, which are rapidly exchanged with the surrounding environment. When native folding is not reached, JUNQ substrates are ubiquitinated and recruit proteasome components, triggering protein degradation. In an important manner, when these mechanisms fail or are diminished, protein misfolding has been observed by the formation of amyloid fibrils [152,153]. In parallel, diverse strategies have evolved to maintain the native structure of proteins for prolonged periods of time, and avoiding their conversion into non-functional misfolded structures [154].

Distinct cytoplasmic structures, spatially distant from JUNQ have been observed to contain large and highly insoluble aggregates [150]. These compartments denominated IPODs (insoluble protein deposits) contain insoluble aggregates and amyloid like-fibrils; whereas multiple IPODs can exist at the same time in cytoplasm, only a single JUNQ is found within each cell. These types of compartments, underscore their essential function in sequestering proteins and triaging them to re-establish native protein folding or initiate protein degradation.

While misfolded polypeptides of JUNQ are sequestered in a detergent soluble state, and the aggregated polypeptides are retained in IPOD [142], other compartments have an important function; Q-bodies whose formation and processing depend on the cortical ER. The maturation and clearance of Q-bodies require chaperones Hsp70 and Hsp90 [142].

Biochemical functions of chaperones and their spatial localization within the cell are fundamental to understand folding impairments during pathological states [142,155], and considering the presence of a dynamic relationship between damaged and aggregated proteins, the function of these compartments could be very important to maintain the proteostasis in the first instance, and collectively cell homeostasis [156].

### 3.4. Proteasome, Structure, and Function

Nascent or newly synthesized polypeptides are predisposed to a high quality control process associated to their folding, avoiding the accumulation of anomalous proteins. Most studied systems to maintain proteostasis, are performed by molecular chaperones, as well as two mechanisms of protein degradation, the ubiquitin-proteasome system and the lysosomal proteolysis through autophagy [157,158,159]. Proteasome (26S) is a multimeric complex whose function is protein degradation through its endoprotease activity. Proteasome acts primarily on short-lived proteins with regulatory functions and on misfolded proteins. Protein degradation is a specific and efficient process, which depends on ATP. It is involved in functions such as modulation of cell cycle, apoptosis, and cell differentiation, response to extreme temperature changes, oxidative stress, immune responses, genetic regulation, and metabolism [160,161].

**Figure 4:**
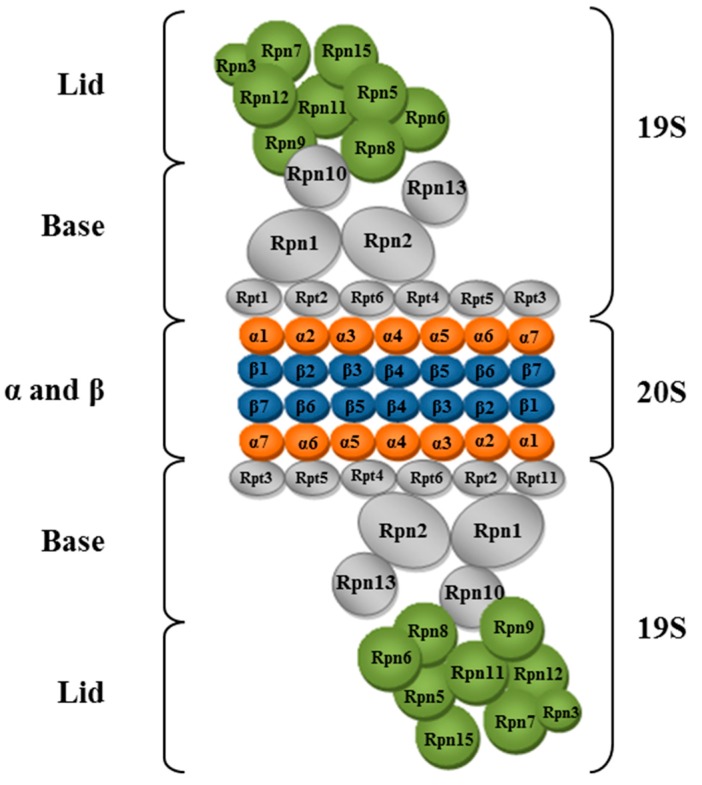
Scheme of the 26S proteasome. Proteins that make up the base and lid of the 19S regulatory subunit are shown. The cylindrical portion of the 20S catalytic subunit is shown in an open conformation, showing the arrangement of α and β proteins identified in orange and blue, respectively (Adapted from [167]).

The 26S proteasome is directly associated to protein degradation via the ubiquitin system. It is composed of a catalytic subunit comprising a cylindrical central structure with proteolytic activity, denominated 20S proteasome. Additionally, two regulatory subunits 19S are present, which have ATPase activity; these are involved in recognition and elimination of ubiquitin chains. Likewise, 19S subunits participate in the unfolding of client proteins [160] (Figure 4).

The 20S catalytic subunit consists of four complexes arranged in the manner of a ring, forming a hollow cylindrical structure. Each ring consists of seven different protein subunits, two rings consist of subunits denominated α (α_1–7_) located at the ends of the cylindrical structure, the other two rings consist of subunits called β (β_1–7_) and are located in the central area [162]. The N-terminal domains of α-subunits occlude access to the interior of the proteasome, while three of the β subunits have a protease activity. Therefore, the 20S subunit is a structure α_1–7_, β_1–7_, β_1–7_, α_1–7_, with proteolytic capacity and highly conserved between eukaryotes [163,164].

The 19S regulatory subunit is a complex consisting of at least 19 protein subunits, which are distributed to form the base and lid of the 19S subunit. The base is formed by six proteins with ATPase activity (Rpt1–Rpt6) that are in contact with α-subunits of the 20S proteasome, and four proteins lacking ATPase activity (Rpn1, 2, 10, 13). The protein subcomplex which makes up the lid is integrated by proteins without ATPase activity (Rpn3–9, 11, 12), but contains binding sites for recognition of ubiquitinated proteins, and maintain a function of deubiquitinase [162,165,166].

The client proteins must have at least four ubiquitins attached to be recognized. Recognition and anchoring occurs through Rpn10 and Rpn13, which associate with polyubiquitin. Rpn11 accomplishes the remotion of ubiquitin chains. Proteins with ATPase activity in the 19S subunit perform the unfolding of ubiquitinated proteins while interacting with the α proteins of the 20S subunit, a condition that allow its opening, and leads to client proteins into the 20S subunit [163,168,169].

Proteolytic activity of the 20S proteasome subunit lies in the extreme N-terminus of the subunits β1, β2, and β5 of β-rings. β1 subunit has a caspase activity on amino acids, β2 shows the trypsin activity on basic amino acids, and β3 subunit a chymotrypsin activity on hydrophobic amino acids. Even more, *in vitro* experiments have shown that the 20S subunit alone can present proteolytic activity, generating peptides of 3–15 residues [167].

Alzheimer’s, Parkinson’s, and Huntington’s diseases are characterized by dysfunction of the ubiquitin-proteasome system, and accumulation of misfolded proteins in the central nervous system. Particularly, in Alzheimer’s disease, the patient has two important lesions, extracellular amyloid plaques, and intraneuronal neurofibrillary tangles formed by Aβ peptide [170,171], which is generated through sequential cleavage of amyloid precursor protein (APP). *In vitro* experiments showed that Aβ40 directly binds to the inside of 20S proteasome and selectively inhibits its chymotrypsin-like activity [172,173]. More recent evidence shows that Aβ42 also impairs proteasome activity [174,175]; both of them may be endogenous inhibitors of the proteasome. This condition is a consequence of dysfunction of the ubiquitin-proteasome and its possible association to disease.

### 3.5. Autophagy Mechanism

Autophagy includes a lysosomal degradation pathway, in which cells self-digest their own components, and has been shown to be essential for survival, differentiation, development, and homeostasis. Autophagy involves the sequestration of cytoplasmic components in double membrane autophagosomes, wherein these structures fuse with lysosomes and their cargoes, and are delivered for degradation and recycling [176].

Mechanisms of autophagy play an important role in removing protein aggregates and organelles that fail to be degraded by the ubiquitin-proteasome system [177]. Furthermore, the role of autophagy in maintaining macromolecular synthesis and ATP production is likely a critical mechanism underlying its evolutionarily conserved pro-survival function [178]. For instance, when cells suddenly undergo a surge in metabolic demand, autophagy may be needed to generate sufficient intracellular metabolic substrates to maintain energy levels. This self-digestion process not only provides nutrients to maintain cellular functions during fasting, but can also relieve cells of superfluous or damaged organelles, misfolded proteins, and invading microorganisms [179]. Indeed, it has been described that autophagy could provide an adaptive role to protect organisms against diverse pathologic conditions; which include: cancer, neurodegeneration, aging, and heart disease [180].

Through this process, cells carry out double-membrane vesicles, denominated autophagosomes, which could sequester organelles, proteins, or portions of the cytoplasm for delivery to the lysosomes [159]. The core pathway of mammalian autophagy begins with the formation of an isolation membrane (also called a phagophore), and involves a minimum of five molecular components, including (1) the AuTophaGy related 1 (Atg1)/unc-51-like kinase (ULK) complex; (2) the Beclin 1/class III phosphatidylinositol 3-kinase (PI3K) complex; (3) two transmembrane proteins, Atg9, and vacuole membrane protein 1 (VMP1); (4) two ubiquitin-like protein conjugation systems (Atg12 and Atg8/LC3); and (5) proteins that drive fusion between autophagosomes and lysosomes. It has been described that some of these core autophagy pathway components are directly modulated by cellular stress signals [181,182].

Several functions of autophagy, such as, elimination of defective proteins and organelles, prevention of protein aggregate accumulation, and clearance of large poly-ubiquitinated proteins, overlap with those of the ubiquitin-proteasome system; however, pathways leading up to autophagy are uniquely capable of degrading entire organelles such as mitochondria, peroxisomes, ER, as well as intact intracellular microorganisms [183]. Unlike proteasomal degradation, the autophagic breakdown of substrates is not limited by steric conditions, because substrates do not need to be unfolded to pass through the narrow pore of the proteasomal barrel. Oligomeric and aggregated proteins are poor substrates for proteasomal degradation, and better targets for autophagic degradation; therefore, preventing the intracellular accumulation of misfolded proteins and contributing to the proteostasis [183].

Autophagy is also upregulated when cells undergo remodeling events, such as developmental transitions or to rid themselves of damaging cytoplasmic components, during oxidative stress or infections [182]. Likewise, autophagy is activated as an adaptive catabolic process in response to different forms of metabolic stress, including growth factor depletion and hypoxia [183]. Through autophagy, bulk degradation generates free amino acids and fatty acids that can be recycled or further processed to maintain ATP production in cells when it is required [182].

Nevertheless, alterations in autophagy could result in the accumulation of ubiquitinated and aggregated proteins, and in turn damaged organelles. In experimental diseases, the self-cannibalistic or, paradoxically, even the prosurvival functions of autophagy may be deleterious [182]. Autophagosomes have been observed to accumulate in the brains of patients with diverse neurodegenerative diseases, including Alzheimer, transmissible spongiform encephalopathies, Parkinson, and Huntington [182]. For instance, autophagosomes accumulate in dystrophic neurons of Alzheimer’s disease patients, possibly as a result of impairment in autophagolysosomal maturation, consequently contributing to the accumulation of pathogenic Aβ peptide [184].

On the other hand, the pharmacological activation of autophagy reduces the levels of soluble and aggregated conformations of mutant huntington, mutated proteins in spinocerebellar ataxia, as well as mutant forms of α-synuclein and tau. This activation reduces cellular toxicity and their neurotoxicity in mouse and *Drosophila* models [185]. Particularly in these models, neuroprotection modulated by autophagy may be due to a quantitative reduction in the amounts of toxic protein species as well as anti-apoptotic effects [185].

Autophagy can influence life and death decisions of cells, being cytoprotective or self-destructive; and being directly linked to apoptotic death pathways. Based on the knowledge of physiological functions of autophagy, it has been determined that both, basal levels of autophagy and stress-induced increase of autophagy, are likely determinant in mammalian homeostasis [186].

## 4. Clinical Focus

Several regulatory and control strategies have evolved in biological systems to protect the phenomenon of protein folding. Molecular chaperones, protease activities, and molecular factors work together to refold or remove proteins [187]. When these defensive housekeeping systems of cells are unable to counteract with these challenges and homeostatic systems are gradually deteriorated, pathological conditions associated with misfolding become evident [11,90,188]. Even without genetic defects, protein translation is sufficiently error-prone to allow a missense mutation in proteins every 1000 to 10,000 amino acids, resulting in defects between 4% and 36% of all new proteins synthesized [189,190,191]. This can be tolerated if these proteins can be degraded, but when the load is excessive, as occurs during cell stress, cell death could appear [192,193].

### 4.1. Neurodegenerative Diseases

Mechanisms preventing amyloid fibril formation are associated with properties of cell environment, including the location of proteins within specific compartments [150,194], as well as the presence of molecular chaperones and degradation mechanisms, such as the ubiquitin–proteasome system and autophagy [195,196,197,198,199] (Figure 2). Protein misfolding is developed when proteins are unable to attain or maintain their biologically active conformation [187].

Protein misfolding and formation of toxic aggregates, for instance could affect the proteostasis in cells to induce ER stress, and in this condition, UPR is required. Likewise, molecular chaperones can target specific steps in the process that leads to misfolding, specifically inhibiting either primary or secondary nucleation processes [200], such as the case of the Hsp70 function [201].

The disturbance of proteostasis can lead to a situation that is considered a metastasis in proteins, therefore initial aggregation events trigger a cascade of pathological processes that could mark the progression of chronic-degenerative diseases [202]. In cases such as Aβ peptide, α-synuclein, and others peptides, the direct connection between misfolding and the formation of amyloid fibrils, is determined by structural transitions at the molecular level [40]. Therefore, amyloid formation is triggered when the protective mechanisms have been exceeded, or due to malfunction of mechanisms of cell regulation [40].

Protein aggregation and amyloid formation are two fields that have been extensively studied by the association of amyloid deposition with a range of chronic degenerative disorders, from Alzheimer’s disease (AD) to diabetes mellitus type 2, many of which are major threats to human health and welfare in the modern world [203,204]. AD is characterized by cognitive alterations, memory loss, and behavioral changes. Amyloid plaques and neurofibrillary tangles are the hallmark lesions in the pathology and both arise from protein misfolding phenomena [205]. In this condition, the Aβ peptide and tau protein suffer conformational changes that span disordered states and lead to maturation of toxic aggregates. The presence of such structures leads to ER stress, activating IRE1 and PERK pathways that active CHOP, conditions reported to induce neuronal death [120]. Likewise, amyloid precursor protein (APP) and presenilins 1 and 2 have been associated with a familiar form of Alzheimer. Taking into account their ER membrane location, stress conditions may alter the activity of presenilin 1 and 2, inducing an increase in the processing of Aβ peptide [206]. In Parkinson disease, the death of dopaminergic neurons and protein aggregation (Lewy bodies) in different regions of the brain, is present [207].

### 4.2. Metabolic Diseases

ER stress is associated with inflammatory and stress signaling pathways, which could exacerbate metabolic dysfunction, contributing to obesity, insulin resistance, fatty liver, and dyslipidemia [208,209]. The presence of amyloid structures in pancreatic islets of Langerhans is a pathophysiological condition related with diabetes mellitus type 2. These deposits are composed of a peptide hormone named amylin [210]. Amylin is normally soluble, and its structure in the monomeric state is natively disordered. However, secondary structure transitions can be important to attain the three-dimensional structure found in amyloid fibrils. Aggregation of amylin is associated with an increased response of ER stress, which leads to dysfunction of pancreatic β cells, apoptosis, and eventually the loss of the cell mass of islets [211,212,213,214]. Likewise, high levels of plasmatic non-esterified fatty acids can contribute to β cell dysfunction [215]. In our work group, we have been able to establish a potential relationship of interaction between these important biomolecules [216].

UPR is chronically activated in atherosclerotic related cells, particularly on advanced lesional macrophages and endothelial cells [217]. Oxidative stress, oxysterols, high levels of intracellular cholesterol, and saturated fatty acids, are conditions that can lead to prolonged activation of the UPR in advanced lesions. Likewise, these arterial wall stressors may be associated with obesity, insulin resistance, and diabetes, all of which promote the clinical progression of atherosclerosis. The potentially important proatherogenic effect of prolonged ER stress is activation of inflammatory pathways. Even more, prolonged ER stress triggers apoptosis in macrophages, which in turn leads to plaque necrosis if the apoptotic cells are not rapidly cleared [217].

Conditions associated to atherosclerosis, such as chronic ER stress, affects systemic risk factors at the level of hepatic lipid metabolism and pancreatic β-cell function [218]. A specific focus has been performed on signaling modulation through IRE1. Recent developments in understanding how IRE1α functions to promote cell death *versus* cell survival at a protein structural level, raise the possibility of several specific drugs that can block IRE1α-dependent cell death [219,220].

### 4.3. Cancer and Protein p53

Factors which contribute to a significant increase in protein misfolding incidences are mutations, thermodynamics, and external stress conditions [187]. The proposal that ER stress signaling could either be beneficial for tumor growth or play a guardian role to prevent cell transformation is very important to analyze [221]. Tumor cells are often subjected to major molecular changes due either to transformation-dependent metabolic demand or to stressful environments, including hypoxia, nutritional stress or pH stress [222]. For example, one of the conditions of activation of UPR in cancer, has been attributed to the hypoxic condition in the tumor surrounding environment [223].

Involvement of PERK and IRE1 arms of the UPR in tumor growth has been broadly characterized [222]. In these conditions, ER stress signaling represents an important constituent of tumor progression and survival [222]. IRE1α enhances angiogenesis and may alter cell adhesion and migration through regulated IRE1‑dependent decay (RIDD). Likewise, cells deficient in XBP1 or PERK have a large reduction in their ability to form solid tumors in mice models. In fact, negative regulation of chaperone activity has been investigated as an anticancer strategy [224,225].

Expression of components of the ER protein-folding machinery, such as BiP, has also been suggested to promote tumor progression, cell survival, metastasis, and resistance to chemotherapy [226]. Strategies to downregulate BiP in models of cancer or through the use of inhibitors of the ATP-binding domain have great cytotoxic potential [227,228,229,230,231,232].

On the other hand, p53 is a transcription factor with an essential role in guarding cells responses to various stress signals, through the induction of cell cycle arrest, and apoptosis as well as effects that are independent of its ability to transactivate gene expression [233]. Mutation of the tumor suppressor p53 is the most frequent genetic alteration in human cancer [234]. The majority of the mutations occur in the DNA-binding domain of the p53 (residues 102–292), which result in loss of DNA binding.

Zinc binding, coordinated by H_179_, C_176_, C_238_, and C_242_, is critical for maintain the native folding of p53 and requires reduction of thiol groups on cysteines. Residues from the loop-sheet-helix motif interact in the major groove of the DNA, while an arginine from one of the two large loops interacts in the minor groove. Loops and the loop-sheet-helix motif represent the conserved regions of the core domain, and contain the majority of the p53 mutations identified in tumors [235]. In this sense, several mutations induce conformational changes in the DNA binding surface [236] although destabilized mutants of p53 can be stabilized by the binding of other molecules [237,238].

Consider that cellular and extracellular spaces are highly saturated environments that allow a wide variety of interactions between molecules. This feature, often referred to as macromolecular saturated environment could have important consequences in the thermodynamics of molecules, affecting the conformational states of proteins [239], and then proteostasis. Even more, according to scientists working in different fields of knowledge, nature appears to have employed disorder to create high levels of organization. In some cases nature seems to have created disorder, when there is, in the first place a lack of it [240]. This situation extrapolated to the role of proteins and their association with disease, could find their origin in the way proteins carry out many structural changes, employing finely tuned disorder-to-order transitions [34].

## 5. Protein Folding in Drug Development

In all organisms, energy and nutrient management requires the highly regulated and coordinated operation of many homeostatic systems. Much of the development and evolution of these systems has taken place in a different environment to the one we now experience as modern humans, which includes excess nutrients, new dietary components, lack of physical activity, and an increased life span [209]. In fact, the requirements for the timespan as well as the magnitude of adaptive responses have dramatically increased due to rises in life expectancy and a chronic lifetime exposure to the stress signals [209].

Opportunities for the development of effective therapies against protein-aggregation disorders lie in the discovery of molecules that decrease the concentrations and formation rates of anomalous protein assemblies or that enable our natural defenses to maintain their efficacy for longer periods of time [40]. A key milestone in the development of any new therapy is the selection of appropriate molecular targets.

### 5.1. Strategies Focus on Amyloidosis

Pharmacological strategies for effective therapy in the treatment of diseases associated with protein folding, might consider the following conditions: inducing the stabilization of native state, reducing the concentration of aggregation-prone species, blocking the nucleation and growth of aggregates; in general conditions that are able to reduce the risk of aggregation. Another strategy is optimization of cell defense mechanisms to maintain their efficacy for longer periods of time, using molecules that can act as pharmacological chaperones. As already suggested, the most effective procedures for the prevention and treatment of misfolding diseases, are likely to be those that address the earliest events in their development [92,241].

Stability of proteins and design of sequences that can efficiently acquire a globular structure, have been considered to be one of the factors that prevent the conversion of a globular protein into amyloid-like fibrils. It has been demonstrated that loss of stability in the native state is a primary mechanism by which mutations promote their pathogenic effects in some hereditary forms of amyloidosis [242]. For example, nature has used strategies to reduce the number of patterns that favor the formation of β-structure, alternating groups of hydrophobic and hydrophilic residues, as well as maintain a high net charge in the sequence, place strategically charged amino acids through the sequence, generate short β-strands on the edges of large β-sheets, incorporate proline residues, and cover β-sheets with α-helices [81,83,243,244].

A representative example in this topic is the drug tafamidis (Fx-1006A), a small molecule that acts as a pharmacological chaperone of transthyretin (TTR), stabilizing the native TTR and some variants. TTR tetramer dissociation has been described as the rate-limiting step in the amyloid formation cascade. Tetramer dissociation is followed by dimer dissociation yielding unstable monomers. TTR monomers easily unfold leading to spontaneous self-assembly into amyloid fibrils [245]. Tafamidis is the first disease modifying pharmacological treatment available to treat familial amyloid polyneuropathy. In fact, fibril formation has been demonstrated as a mechanism for sequestration of oligomeric species, in a way that cells reduce toxicity [246].

### 5.2. Chemical Chaperones

Identification of molecule regulators of the UPR signaling as potential therapeutic strategies to treat protein misfolding and other human diseases, results in a promising approach. UPR is considered a target for drug discovery because of emerging evidence from animal models indicating its contribution to diverse diseases, including cancer, metabolic diseases, diabetes, neurodegenerative disorders, inflammation, liver dysfunction, and brain and heart ischaemia [247].

Development of drugs that interfere with ER stress, have wide therapeutic potential. Five groups of strategies according to their mechanism of action have been characterized: compounds directly binding to ER stress molecules, chemical chaperones, inhibitors of protein degradation, antioxidants, and drugs affecting calcium signaling. Treatments are generally inhibitory, also lead to increased viability, except when applied to cancer cells [248].

Chemical chaperones are described as low-molecular mass compounds that stabilize the folding of proteins and buffer abnormal protein aggregation. In this case, chemical chaperones have been shown to improve ER function, through diminishing protein misfolding events. The most studied chemical chaperones in a disease context are 4-phenylbutyrate (4-PBA) and tauroursodeoxycholic acid (TUDCA), which have been approved by regulatory authorities for primary biliary cirrhosis (4-BPA) and urea cycle disorders (TUDCA) [249].

In animal models of obesity, chemical chaperones reduced ER stress in the liver of mouse, improved insulin sensitivity and glucose homeostasis [250], and reversed leptin resistance [251]. Treatment with 4-PBA also improved glucose tolerance in patients with insulin-resistance [252], and TUDCA partially restored insulin sensitivity in liver and muscle [253].

High-throughput screening for IRE1 modulators has identified plant-derived flavonols as activators of IRE1 sensors, as well as possible new regulatory sites of interaction. Docking of small-molecule libraries suggests the presence of a pocket associated with dimerization/oligomerization of IRE1 including a binding site to sterols, which could represent an important binding site for the regulation of IRE1 signaling [254,255]. Comparative and systematic studies are needed in a better way to define the real therapeutic value of manipulating ER stress levels, in addition to outlining possible side effects [209].

Likewise, approaches through compounds that bind and stabilize mutants of p53 have been performed. Upon screening of a library of over 100,000 compounds and further optimizing of the hits, compound 7 (CP-31398) was shown to promote the conformational stability of wild-type p53 DNA-binding domain and that of full-length p53 [237,256]. Screening of the diversity set from the National Cancer Institute led to the discovery of some chemical chaperones with mutant p53-reactivating capacity: compound 854 known as PRIMA-1 (p53 reactivation and induction of massive apoptosis) and compound 954 known as MIRA-3 (mutant p53 dependent induction of rapid apoptosis). Additional screening of small compound libraries also identified compound 1055 denominated STIMA-1 (SH group-targeting compound that induces massive apoptosis) [187].

### 5.3. Final Considerations

Three-dimensional structure of proteins in general, and specifically proteins that participate in UPR could provide critical information for the development of new pharmacological treatments, this approach may be incomplete, since the disordered domains of proteins involved in UPR and chaperones, have been proven to be critical for their function. Likewise, complex pathways to ensure proteostasis in different subcellular compartments, defined as unfolded protein responses have evolved in the cytosol and mitochondria, which are finely coordinated and require close communication with the nucleus [248]. Even more, the UPR has demonstrated impact on various immune cells, in which it regulates the secretion of pro-inflammatory cytokines and innate immunity signals [254]. These conditions reflect the complexity of cell physiology that might be considered for drug development.

Whereas cellular and extracellular spaces are highly saturated environments [97,98] which allow a wide variety of interactions between molecules, not all biologically active compounds have the desired physicochemical properties to be a drug, which must be sufficiently lipophilic to be absorbed, maintain polar properties to cross the gastrointestinal wall, and have a vulnerable chemical functionality, then molecules can be targeted by liver catabolic systems [26]. Without doubt a more complex understanding of the threshold of responses that occur within cells to sustain the proteostasis, implies a greater understanding of the regulatory mechanisms that regulate protein folding, which will result in an increase in tools that could be the basis to modulate the functional activity of therapeutically important proteins.

## 6. Conclusions

Mechanisms that modulate protein folding within the highly saturated cellular environment, which are regulated by a highly sophisticated network of communication between proteins, reflect the complexity of cellular processes. These features span several molecular hierarchies, from the use of small disordered regions within intricate three-dimensional structures, and an effective folding phenomenon to keep hidden the hydrophobic domains. Likewise required are the activity of highly organized molecules such as chaperones, and the participation of pathways associated with complete degradation of organelles, to maintain the homeostasis of proteins as a whole. Evidence indicates that nearly all large, subcellular processes, from the organelles to ribosomes, may have specific ways of sensing the proteome and reacting to proteotoxic stress, and so that every step in the life of proteins is under close scrutiny. Therefore, the insights into the features of the functional conformations of proteins, the environments in which they work, and the ways that cellular defense mechanisms normally function so effectively together to maintain protein homeostasis, can expand the possibilities for better treatments against human diseases.
